# 1749. Nursing Home Providers’ Empiric Antibiotic Choices for Residents with Urinary Tract Infections: A National Survey

**DOI:** 10.1093/ofid/ofac492.1379

**Published:** 2022-12-15

**Authors:** Lindsay Taylor, Jessica Irvine, Brigid Wilson, Justin Massey, Mriganka Singh, Sally Jolles, Corinne Kowal, Taissa A Bej, Jon P Furuno, David Nace, Robin L Jump, Christopher J Crnich

**Affiliations:** University of Wisconsin School of Medicine and Public Health, Madison, Wisconsin; University of Wisconsin School of Medicine and Public Health, Madison, Wisconsin; Louis Stokes Cleveland VA Medical Center, Cleveland, Ohio; University of Wisconsin School of Medicine and Public Health, Madison, Wisconsin; Alpert Medical School, Providence, Rhode Island; University of Wisconsin School of Medicine and Public Health, Madison, Wisconsin; Louis Stokes Cleveland VA Medical Center, Cleveland, Ohio; Louis Stokes Cleveland VA Medical Center, Cleveland, Ohio; Oregon State University, Portland, Oregon; University of Pittsburgh School of Medicine, Pittsburgh, Pennsylvania; University of Pittsburgh School of Medicine, Pittsburgh, Pennsylvania; University of Wisconsin School of Medicine and Public Health, Madison, Wisconsin

## Abstract

**Background:**

Urinary tract infections (UTIs) are the most common indication for antibiotic prescriptions in nursing homes (NHs) and frequently result in fluoroquinolone (FQ) prescriptions. We performed a vignette-based survey of NH providers to better understand empiric UTI treatment decision-making.

**Methods:**

Study participants were recruited nationally through professional organizations and snowball sampling from December 2021 to February 2022. Clinical vignettes depicting four UTI presentations in NH residents (1. simple cystitis, 2. pyelonephritis with cephalosporin allergy, 3. catheter-associated UTI and 4. cystitis with history of resistant organism) were developed and distributed via electronic survey. Respondents provided free-text antibiotic choice, which two physicians independently reviewed and implicitly determined if a preferred or not-preferred antibiotic was selected. A panel of three physicians adjudicated discrepancies between the primary reviewers. Analysis was performed in R.

**Results:**

Of 86 respondents, 74% were physicians and 26% were advanced practitioners. Half of respondents (50%) had >10 years NH experience, 41% were geriatrics trained, and none were infectious disease trained. Figure 1 details antibiotic choices and preferred agents for each case. Overall, 70% of antibiotic choices were deemed preferred antibiotics, with the least number of preferred choices observed for case 3 depicting catheter-associated UTI (53%). FQs (43%) and nitrofurantoin (14%) were the most frequent non-preferred choices. Case 2 received the greatest proportion of FQ prescriptions (38%), but this was a preferred agent. In cases where FQs were not a preferred choice, they comprised 17% of antibiotic choices. There was no difference in FQ or preferred prescribing choices by role. Providers with >10 years NH experience, however, prescribed fewer FQs over the four cases than those with less NH experience (0.7 vs. 1.1, p=0.04).

Antibiotic Choice and Preferred Agents per Case

**Figure:**
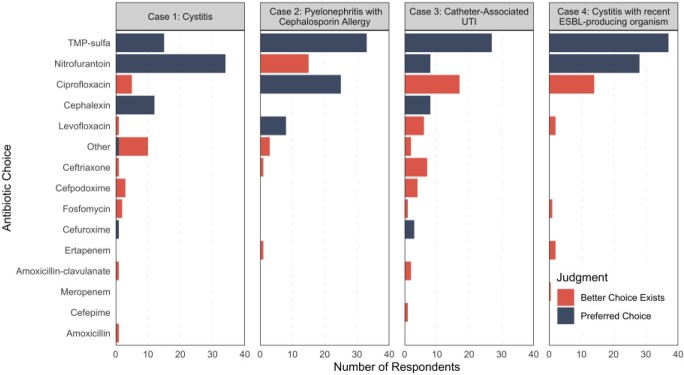

Antibiotic choices are ordered from highest to lowest cumulative frequency.

**Conclusion:**

This sample of NH providers made mostly preferred empiric antibiotic choices, however, FQ use remained high, particularly in providers with less NH practice experience. Further exploration of decision-support tools for empiric antibiotic prescribing in this setting may improve antibiotic stewardship.

**Disclosures:**

**Robin L. Jump, MD, PhD**, Merck: Grant/Research Support|Pfizer: Advisor/Consultant.

